# Cortical mechanisms for afterimage formation: evidence from interocular grouping

**DOI:** 10.1038/srep41101

**Published:** 2017-01-23

**Authors:** Bo Dong, Linus Holm, Min Bao

**Affiliations:** 1 CAS Key Laboratory of Behavioral Science, Institute of Psychology, Beijing 100101, P.R. China; 2 University of Chinese Academy of Sciences, Beijing 100101, P.R. China; 3 Department of Psychology, Umeå University, S-901 87 Umeå, Sweden

## Abstract

Whether the retinal process alone or retinal and cortical processes jointly determine afterimage (AI) formation has long been debated. Based on the retinal rebound responses, recent work proposes that afterimage signals are exclusively generated in the retina, although later modified by cortical mechanisms. We tested this notion with the method of “indirect proof”. Each eye was presented with a 2-by-2 checkerboard of horizontal and vertical grating patches. Each corresponding patch of the two checkerboards was perpendicular to each other, which produces binocular rivalry, and can generate percepts ranging from complete interocular grouping to either monocular pattern. The monocular percepts became more frequent with higher contrast. Due to adaptation, the visual system is less sensitive during the AIs than during the inductions with AI-similar contrast. If the retina is the only origin of AIs, comparable contrast appearance would require stronger retinal signals in the AIs than in the inductions, thus leading to more frequent monocular percepts in the AIs than in the inductions. Surprisingly, subjects saw the fully coherent stripes significantly more often in AIs. Our results thus contradict the retinal generation notion, and suggest that in addition to the retina, cortex is directly involved in the generation of AI signals.

After fixating on an image for a period of time, an illusory percept in complementary luminance and colors can be observed when the original inducing image is removed. This is called negative after image (AI). AI formation has traditionally been attributed to the bleaching of retinal photoreceptors^1^,^2^.

More recent work proposes that AI formation can also be affected by some perceptual and cognitive factors. For example, it has been found that AI of a pattern becomes weaker when the pattern is attended^3^,^4^,^5^. Moreover, the perceived size of the inducing stimulus determines the size of the AI^6^. Furthermore, adaptation to perceptual filing-in of a surface leads to AI of such a surface^7^. AI is also affected by contours. For instance, color appearances in AI can spread to regions not previously adapted to color, and are triggered and constrained by contours presented after the inducing stimulus^8^. Furthermore, sharp luminance edges enhance AIs more than they enhance physical stimuli of similar appearance^9^. Binding appears generally relevant in AI formation, but more strikingly, misbinding of visual features may happen in AI. For instance, color and form may be misbound in AI when misbinding is perceived during the induction phase^10^. Furthermore, several studies suggest that perceptual awareness affects AI. For instance, when the inducing stimuli are suppressed from awareness, the stimuli produce weaker AIs^5^,^11^,^12^. Additionally, previously suppressed percepts initially dominate perception in AI rivalry^11^,^13^.

All the work listed above, covering the topics of attention, size perception, perceptual fill-in, contextual modulation, feature binding and awareness, consistently supports the hypothesis that AI formation is not exclusively generated by retinal mechanisms, but that cortical processes are also involved in determining the AIs. We call this hypothesis the cortical generation notion. Importantly, the cortical generation notion also admits the contribution of the retina in AI formation.

Recent work by Zaidi and colleagues, however, casts doubt on the cortical generation notion^14^. Zaidi and colleagues found that after the removal of the inducing stimuli, the ganglion cells generated rebound responses that could provide AI signals for later neurons. Accordingly, in their abstract, they conclude that “*afterimage signals are generated in the retina, but may be modified like other retinal signals by cortical processes, so that evidence presented for cortical generation of color afterimages is explainable by spatio-temporal factors that apply to all signals*”.

In their discussion, Zaidi and colleagues^14^ explain this view in more detail; “Because to thalamic and cortical cells, spikes transmitted as part of retinal rebound signals are no different from any other spikes from the retina, cortical processes, such as simultaneous contrast and selective attention, should be expected to modify afterimage signals. Thus, the visually striking demonstrations of these modifications may require no new mechanisms for their explanation. Similarly, retinal rebound signals should generate filling-in under the same spatial and temporal conditions as other retinal signals … Consequently, although cortical adaptation is responsible for many aftereffects, e.g., motion and tilt, our results make it unlikely that it generates color afterimages to prolonged viewing of moderate lights.” In other words, Zaidi *et al*. advocate that AI signals are exclusively generated in the retina. We henceforth call their position the retinal generation notion and point out that it stands in direct contrast to the cortical generation notion.

Evidently, Zaidi *et al*.’s^14^ work makes a strong case for the retinal generation notion by demonstrating that AI signals can be generated in the retina through the adaptation of retinal ganglion cells. However, adaptation affects responses at several stages of the visual hierarchy (for a review see ref. 15). This raises the question as to whether cortical adaptation may also contribute to AI formation? It seems difficult to reach an affirmative answer to the question from the previously reviewed studies which advocate cortical generation of AIs^5^,^7^,^8^,^10^, since most of those studies only investigated how perceptual or cognitive factors *modulate* AIs. Modulation effects alone can be parsimoniously explained by the retinal generation notion (see the cited work by Zaidi and colleagues presented above). Nevertheless, the success of the retinal generation notion in accounting for the effects demonstrated in those studies does not imply that the cortical generation notion is necessarily wrong and should be abandoned.

The present study sought to test the validity of the cortical generation notion more directly by introducing a novel effect of interocular grouping in AIs that contradicts the prediction of the retinal generation notion. In logic, this method is called *reduction to absurdity* and is also known as “indirect proof”. The routine steps in “indirect proof” is to first assume that the opposite of what you are trying to prove is true, and then seek a particular case for which predictions derived from this assumption contradict with the actual observations. The special case for the present work to test the retinal generation notion is a phenomenon serendipitously noticed in one of our studies. We observed that the percepts of complete interocular grouping were more prevalent for the AIs than for the inductions. This phenomenon is described in detail in Experiment 1. Our Experiment 2 ruled out some alternative explanations for the findings in Experiment 1. Importantly, the results in Experiments 2a and 2b showed that interocular grouping during the inductions was reduced as the inducing contrast increased.

With these two pivotal observations in mind, we may move on to the deduction process of “indirect proof”. In case some readers are not familiar with indirect proof, a math example is described in the Supplementary Information. The deduction process for the present study bears much resemblance to that example. As we note, the present study tries to prove the cortical generation notion. Thus, we would first assume that the retinal generation notion is true. Generally, AIs appear after adaptation to a stimulus, and adaptation reduces the gain of the visual system. That is, the visual system is not in the same state during AIs as it is during the presentation of a stimulus with AI-similar contrast. Assuming that the retinal generation notion is correct, i.e. the retina is the only origin of AI signals, comparable contrast appearance would require stronger retinal signals in the AIs than in the inductions with AI-similar contrast. According to the findings in Experiment 2 that increased inducing contrast caused decreased interocular grouping during the inductions, we may reach a prediction that more frequent monocular patterns should be perceived in the AIs than in the inductions with AI-similar contrast. However, this prediction contradicts the empirical observations of subjects actually seeing the fully coherent stripes significantly more often in AIs. As a result, the retinal generation notion is rejected. The present study thus suggests that AI formation should also involve cortical processes in addition to the retinal mechanisms.

## Results

**Figure 1:**
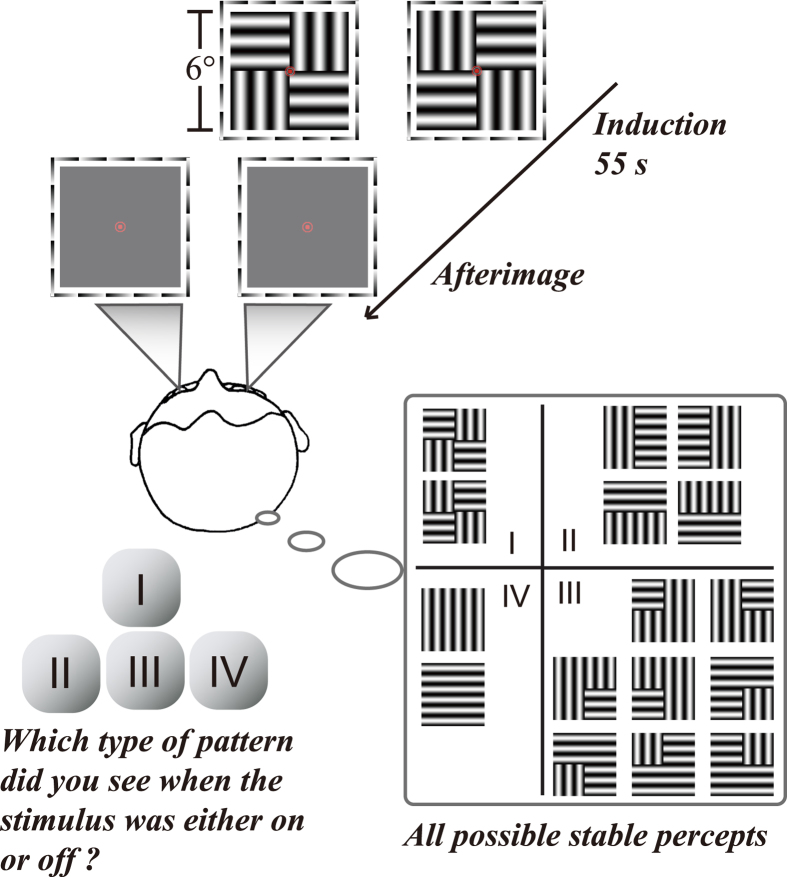
Example stimulus presented to each eye (the top panel), and all possible stable percepts that subjects might experience (the bottom panel). The two checkerboards were rotated 90 degrees with respect to each other, which produced binocular rivalry, and could generate percepts ranging from either monocular pattern (type I) to complete interocular grouping (type IV).

The stimuli we used are shown in Fig. 1. Each eye was independently presented with a 2 by 2 checkerboard of horizontal and vertical grating patches. Each patch of the two checkerboards was rotated 90 degrees with respect to each other (see the top panel in Fig. 1), which produces binocular rivalry, and can generate percepts ranging from complete interocular grouping (perceiving only coherent horizontal or vertical stripes; see type IV in the bottom panel of Fig. 1) to either monocular pattern (perceiving only a checkerboard; see type I in the bottom panel of Fig. 1). Rarely, some subjects perceived the fusion between the left and the right eye image (i.e. plaid). This type V percept was measured in Experiment 2b, though not in our first experiment because it was not noticed at that time.

### Enhanced interocular grouping in negative afterimages (Experiment 1)

In each trial, the full contrast checkerboards were dichoptically displayed for 55 s (*i.e*. induction phase), followed by an AI phase during which all gratings were removed (see the top panel in Fig. 1). During both phases, subjects were told to report which of the four types of patterns they saw by pressing and holding one of the four keys (see the bottom panel in Fig. 1). Ten naïve subjects participated in this experiment. After a brief practice period, each subject completed 15 trials.

**Figure 2:**
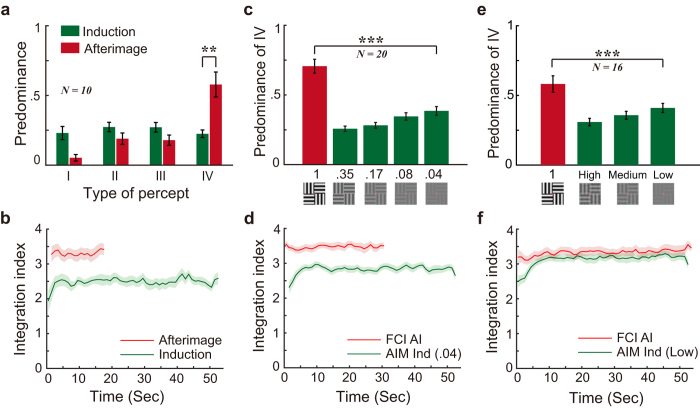
Results of Experiments 1, 2a and 2b. (**a**) Predominance for the four types of percepts during the induction and afterimage (AI) phases in Experiment 1. Predominance represents the proportion of time for one of the four types of patterns perceived. (**b**) Time course of the integration index in Experiment 1. (**c**) Predominance results in Experiment 2a (Multiple inducing contrasts). The green bars indicate the predominance for the coherent percepts (type IV) during the induction of different contrast levels. The red bar indicates the predominance of type IV during the full-contrast-induced (FCI) AI phases. (**d**) Time course of integration index for the FCI AIs and the 0.04 contrast afterimage-mimicking inducers (AIM ind) in Experiment 2a. (**e**) Predominance results in Experiment 2b (Low-pass Filtered Inducers). (**f**) Time course of the integration index for the FCI AIs in Experiment 2b, and the lowest contrast AIM inducers. Error bars and the shaded areas represent standard errors of the means.

AIs lasted for 28.9 ± 14.6 s (*M ± SD*). To calculate the respective predominance during the induction and AI periods, phase durations for each type of percept were summed up across all the trials during the same period. Although all four percept types were perceived approximately equally often during the induction phases (see Fig. 2a, type I: 23.0%, type II: 27.3%, type III: 27.2%, type IV: 22.5%), subjects reported seeing the coherent patterns much more frequently during the AI period than the induction period (57.9% vs. 22.5%; *t*(9) = 3.66, *p* = 0.005, Cohen’s effect size index *d* = 1.16, 95% CI [0.14, 0.57]).

### AI-mimicking induction: multiple inducing contrasts (Experiment 2a)

In our original finding, the contrast of the inducers appeared much higher than those of the AIs. Therefore, the observation of interocular grouping of dichoptic AIs being much stronger than for dichoptic inducers can simply be ascribed to the general behavior of the visual system when receiving faint input signals. To address this issue, we examined interocular grouping during low contrast inductions that mimicked the signal strength of the AIs. The key comparison is between the full-contrast-induced (FCI) AIs and AI-mimicking (AIM) inducers.

In a separate preliminary test, one author and one naïve subject empirically measured the apparent contrast of FCI AIs. Two contrast ramps were displayed during the AI phase, with their inner edges 3.5° away from the fixation point. Two ramps were placed vertically to the left and right sides of the adaptation region. Each ramp consisted of seven gratings (1° × 1°, 1 cyc/deg in spatial frequency) in a row whose contrasts increased logarithmically from 0.01 to 0.64. Since the apparent contrast of AI was relatively high in the beginning and decreased gradually over time, the subjects kept reporting which reference grating resembled the AI most until the AI was no longer visible. Each subject completed 5 trials. The contrast for the point of subjective equality ranged between 0.04 and 0.64 (for details, see Supplementary Table S1 in the Supplemental Materials). Because we hypothesized that lower inducing contrasts would produce more coherent percepts, we selected five contrast levels biased toward the lower end of AI matched contrasts, as well as the full contrast inducer for comparison with Experiment 1 [.04.082.17.35 1]. Given that the grating with 0.04 contrast was too weak to always be perceived, subjects were also instructed to release all the keys when they failed to see anything during the induction.

The duration of AIs generally increased as a function of the inducing stimulus contrast (3.8, 5.2, 7.1, 11.0, and 38.6 s for the five contrasts). Although the distribution of percept types was fairly similar across the inducing contrast conditions (for details see Table 1), subjects did see the coherent patterns slightly more often as the inducing contrast decreased (see Fig. 2c, linear trend analysis, *t*(19) = 5.91, *p* = 0.000011, *d* = 1.32, 95% CI [0.29, 0.61]). This suggests that the interocular grouping was in relation with the strength of the retinal signal such that interocular grouping increased with decreased contrast in the stimulus. Even so, subjects reported seeing the coherent patterns with a much higher probability for the FCI AIs than for the lowest-contrast inducers (70.6% vs. 38.6%; *t*(19) = 5.80, *p* = 0.000014, *d* = 1.30, 95% CI [0.20, 0.44]).

### AI-mimicking induction: low-pass filtered inducers (Experiment 2b)

Eye fixation jitter during induction can blur AIs by shifting the edges of the adaptation region, much like a low-pass filter does^9^,^16^. This may cause the retinal rebound signals to be different during AI compared to induction, *e.g*. blur may cause the AIs to contain more energy at lower spatial frequencies. To control for blur, another AI-mimicking experiment was conducted with low-pass filtered stimulus (see Supplementary Fig. S1).

One may argue that in Experiment 2a, the apparent contrast of FCI AIs were not precisely measured for each individual, but instead estimated according to the pilot test in only 2 subjects. Therefore, in this experiment, the apparent contrast of FCI AIs were measured for each subject in a preliminary test. We found that the apparent contrast for FCI AIs ranged between 0.02 ± 0.01 and 0.42 ± 0.16 (for details, see Supplementary Table S2). Thus three levels of the mimicking contrast were individually selected for each subject: the lowest contrast (0.02 ± 0.01), the highest contrast (0.42 ± 0.16), and the medium contrast (0.10 ± 0.02) which was the square root of the product of the lowest and the highest contrasts. Subjects in this experiment were assigned an extra response key to report seeing the grid patterns (Type V). Besides, if the AI at any quadrant(s) was not visible, subjects were instructed to release all keys.

Subjects perceived the coherent patterns much more often for the FCI AIs than during the lowest-contrast inducers (58.2% vs. 41.0%, paired t-test: *t*(15) = 2.82, *p* = 0.013, *d* = 0.71, 95% CI [0.30, 0.42], see Fig. 2e and more details in Table 1. The durations of AIs were 3.1, 7.4, 16.6, and 49.3 s for the four inducing contrasts). As in Experiment 2a, subjects in Experiment 2b also saw the coherent patterns slightly more often as the inducing contrast decreased (See Fig. 2e, linear trend analysis, *t*(15) = 3.371, *p* = 0.004, *d* = 0.8425, 95% CI [0.0372, 0.1651]).

Over time, the perceived contrast of AIs might decrease below the lowest inducing contrast we employed. This would result in more frequent coherent percepts in AIs and potentially account for the difference in percept type between periods of AI and stimulus induction. Therefore, we performed a time course analysis for both the induction and AI data. In each trial, predominance for the types I-IV was computed for each 1-s time bin. As the percepts of type I-IV corresponded to a general increase of interocular grouping, we estimate the subjects’ interocular grouping tendency over time. For each time bin, the predominance values for the four types of percepts were multiplied with a contrast vector [1 2 3 4]. The sum of the product was defined as the integration index, with larger positive index corresponding to stronger interocular grouping. The integration indices of each time bin was then averaged across trials for each subject.

According to our preliminary test, the apparent contrast of the FCI AIs in the first few seconds was probably 0.42 ± 0.16 (see Supplementary Table S2 for details), which was much higher than the lowest inducing contrast (0.02 ± 0.01). If apparent contrast alone determines the degree of interocular grouping, one would expect weaker interocular grouping during the first few seconds of the FCI AI phases than during the lowest contrast induction. However, the results do not support this notion. Instead, the integration indices during the first 3 s of the FCI AI phases were not significantly different from the asymptote of the integration index during the lowest contrast inductions (paired t-test, *t*(15) = 0.019, *p* = 0.98, *d* = 0.0048, 95% CI [0.396, 0.403], see Fig. 2f), suggesting that interocular grouping during the first few seconds of the FCI AI phases was no weaker than during the lowest contrast inductions.

Furthermore, if the gradual decay of AIs increased interocular grouping over time, one would expect to see an increasing slope in the time course of the integration index in AIs. However, no significant increasing trend was observed within the first 20 s of the time course in Experiments 1, 2a and 2b when induced with full contrast (see the red curves in Fig. 2b,d and f, linear trend analysis: Experiment 1: *t*(9) = 0.67, *p* = 0.519, *d* = 0.21, 95% CI [−21.54, 39.69]; Experiment 2a: *t*(19) = 0.66, *p* = 0.517, *d* = 0.15, 95% CI [−10.86, 20.89]; Experiment 2b: *t*(15) = 1.68, *p* = 0.114, *d* = 0.42, 95% CI [−6.08, 51.23]). Therefore, we believe that the decay of AIs cannot account for the stronger interocular grouping during the AIs than during the AIM inductions.

### Interocular phase alignment (Experiment 2c)

**Figure 3:**
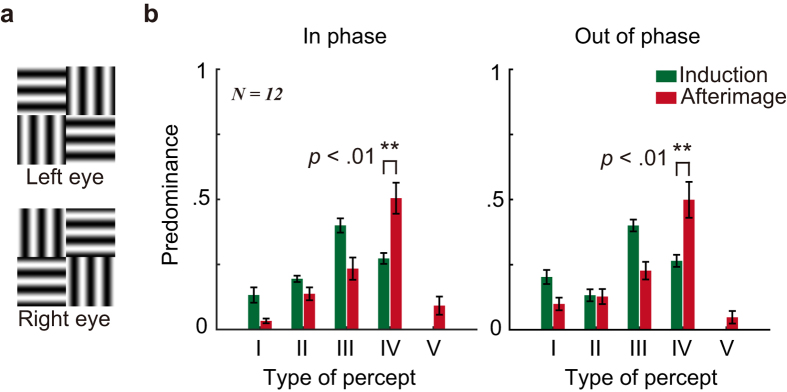
Experiment 2c (Interocular phase alignment). (**a**) Example stimulus of the “out-of-phase” session in each eye. The adjacent grating elements for different eyes were out of phase (e.g. see the upper left element in the left eye and the upper right element in the right eye). (**b**) Predominance for all types of percepts during the induction and afterimage phases. Type V reflects perception of a plaid.

Eye fixation jitter during the induction is never 100% correlated across the two eyes. As a result, the phase alignment of the gratings presented to different eyes will also be unstable. It seems plausible that the stable phase alignment for the interocular patches in the AI condition enhances the effects of interocular grouping for this condition relative to the AIM induction condition. If this is the case, rendering the inducer elements out of phase at the adjacent quadrants in the opposite eye (Fig. 3a) might substantially reduce the proportion of coherent percepts. To answer this question, we conducted Experiment 2c.

AIs lasted for 40.0 ± 14.1 s for the “in-phase” condition and 41.2 ± 38.5 for the “out-of-phase” condition. As shown in Fig. 3b, the two induction conditions produced very similar predominance patterns. A 2 (testing stage: induction vs. AI) × 2 (phase alignment: in-phase vs. out-of-phase) repeated measurement ANOVA on the predominance for the coherent percepts revealed a significant main effect of testing stage (*F*(1, 11) = 13.85, *p* = 0.003, *d* = 1.12), indicating higher predominance of coherent percepts for AIs. However, no significant main effect of phase alignment (*p* > 0.250) or interaction (*p* > 0.250) was found. These results suggest that the mechanisms controlling interocular grouping should arise from polarity independent cells. Therefore, disturbed phase alignment during induction cannot account for the enhancement of interocular grouping for the FCI AI condition relative to the AIM induction condition.

### Eye movements and AIs (Experiment 3)

To further rule out eye fixation jitter accounts of our main findings, we recorded participants’ eye movements in Experiment 3. AIs are stabilized on the retina, but the inducers are never stabilized due to fixational eye movements. One may argue that the interocular grouping during the inductions was less successful than during the AIs because of the retinal stabilization being more disturbed by fixational eye movements during the inductions than during the AIs. In other words, it is not AI per se but a general effect of eye movement that drives our findings. If this is the case, one would observe a close correlation that more eye movements always correspond with less coherent percepts during the inductions. For this purpose, we computed the correlation between the frequency of coherent percepts and the number of blinks, saccades, microsaccades, and lengths of drifts. In the lowest contrast induction condition in Experiment 2b, subject could see the grating during 86.1% ± 14.5% of the total induction period (55 s), and 5 out of 16 subjects saw the grating less than 80% of the time. Therefore, a slightly higher inducing contrast (0.04) was used in this experiment.

To evaluate the stability of fixation, a 2D Gaussian model was fit to the spatial distributions of the gaze positions during the full contrast induction phases. The position of the fit was centered 0.2286° and 0.0972°away from the fixation point (see Supplementary Fig. S6 and Table S4 for details). The width of the fit was 0.9496° (σ_x_) and 0.6907° (σ_y_). These results showed that most subjects maintained steady central fixations (also see Supplementary Fig. S5).

**Figure 4:**
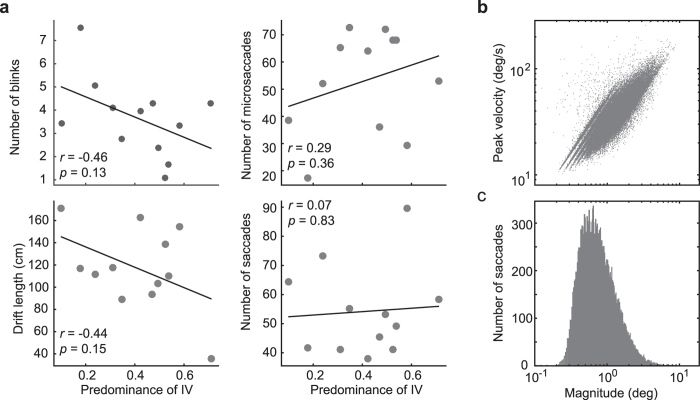
Results of Experiment 3 (Eye movements tracking). (**a**) Correlation between the predominance of type IV percepts and the number of blinks (upper left), microsaccades (upper right), saccades (lower right), and drift length (lower left) across the subjects. Each dot represents a subject. (**b**) Microsaccadic and saccadic peak velocity–magnitude relationship for all subjects combined. Each dot represents a microsaccade or a saccade with peak velocity indicated on the *y*-axis and magnitude indicated on the *x*-axis. (**c**) Magnitude distribution of microsaccades and saccades.

No significant correlation was observed between the extent of interocular grouping during induction and any of our eye movement indices (number of blinks, 16.02 ± 14.72, *r*(10) = −0.46, *p* = .13; saccades, 54.19 ± 15.34, *r*(10) = 0.07, *p* = 0.83; microsaccades, 53.09 ± 18.57, *r*(10) = 0.29, *p* = 0.36; and length of drifts, 117.05 cm ± 37.00 cm, *r*(10) = −0.44, *p* = 0.15, see Fig. 4a). Correlation coefficients for individual participants are shown in Table 2. In only 1 out of 12 subjects, was the extent of interocular grouping reliably inversely related to the number of blinks. Furthermore, two subjects displayed individually positive correlations between number of blinks/microsaccades and the extent of interocular grouping. Also, no significant correlation was observed between the strength of interocular grouping during the full contrast inductions and any of the eye movement indices (see Supplementary Fig. S2, Table S3). Taken together, fixation jitter during induction does not appear to lead to less interocular grouping in the inducers than in the AIs.

We also re-examined whether our selection of the filtered stimuli in Experiment 2b was appropriate. The results were shown in the Supplementary Figure S7. For each trial of each subject in Experiment 3, we estimated the image on the retina at each time point (i.e. 4 ms for the 250 Hz sampling rate of the eye movement recording) based on the x y coordinates of the eye fixations. These images were superimposed on each other to simulate the blurred adaptation region on the retina. We then performed a pixel-by-pixel correlation analysis between this simulated image and each of the nine possible candidates shown in the Supplementary Figure S1. Specifically, each image array was reshaped into an N by 1 vector. We thereafter ran a Pearson’s correlation analysis between every pair of vectors to obtain a 3-by-3 array of the correlation coefficients for the nine candidates. For each subject, the arrays for the 15 trials were averaged to show the average correlation coefficients for the 9 candidate patches. The results of the correlation analysis suggested that we could use either candidate filter in the left two columns of the 3-by-3 array. The filter we actually selected in Experiment 2b happened to be one of them.

## Discussion

The present study reports a novel phenomenon that interocular grouping is more prevalent in AIs as compared to inducers. By the comparison with the inducers with AI-similar contrast, the results of Experiments 2a and 2b suggest that lower contrast and blurry appearance of the AIs are not sufficient to account for the enhanced interocular grouping during the AIs. Since AIs are stabilized on the retina, but the AIM inducers are not, the enhanced interocular grouping for the AIs might simply be due to the distinct contribution of eye movements between the two conditions. The results of Experiment 2c suggest that interocular grouping is not dependent on the interocular phase alignment. Therefore, the interocular phase misalignment in the AIM induction condition is not likely to strongly affect the degree of interocular grouping. Experiment 3 further explored the relation between the prevalence of interocular grouping and eye movements. Because no significant correlation was found between the eye movement measures and interocular grouping, our observed effects do not appear to be due to unbalanced influences of eye movements on the AIs and inductions. However, caution is warranted when making a conclusion based on negative results. Therefore, more extensive future work is needed to fully test the eye movement argument.

Our work lends new support to the hypothesis that AI formation at least partially results from cortical adaptation. The results also go against predictions derived from Zaidi *et al*.’s retinal generation account which hold that interocular grouping in FCI AIs should be no stronger than during the presentation of AIM inducers. The FCI AIs and AIM inducers are similar in local appearance, and therefore, their respective cortical output signals are presumably similar in strength^17^,^18^,^19^. Following adaptation to full contrast, neural gains of the thalamic and cortical cells in the FCI AI condition are substantially reduced^20^,^21^. However, this is not the case during the AIM induction for which the adapting contrast is low^22^. If the retina is the only origin of AI signals, as stated in Zaidi *et al*.’s retinal generation account, comparable cortical outputs between the two conditions would require stronger retinal signals for the FCI AIs than for the AIM inducers. However, a stronger retinal signal should if anything lead to stronger monocular neuronal activities in the FCI AI condition (for simplicity, the thalamic signals are not discussed).

**Figure 5:**
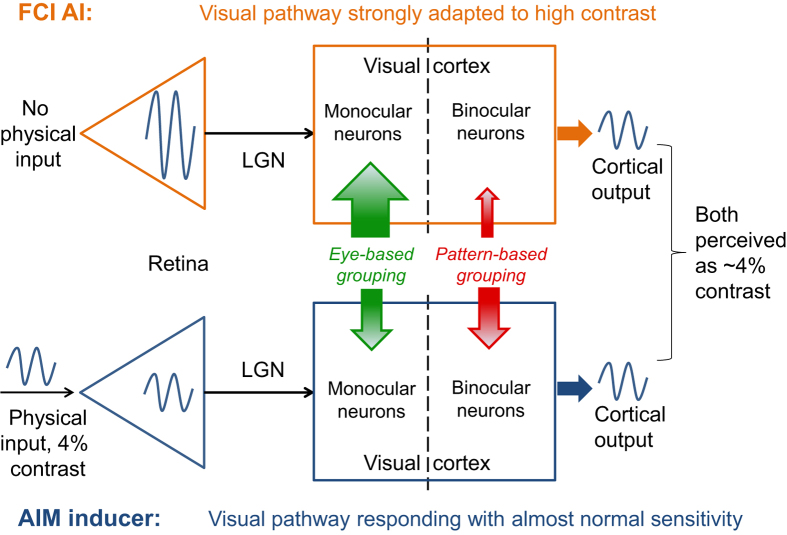
Schematic diagram showing the retinal and cortical activity profile for the FCI AI (upper) and AIM inducer (lower) conditions. Based on the assumption that the retinal generation notion is true and the results in Experiment 2 (lower contrast producing more interocular grouping) hold, one would predict that more frequent monocular patterns would be perceived in the FCI AIs than in the AIM inductions, as denoted by the larger green arrow and smaller red arrow for the FCI AI condition than for the AIM inducer condition. However, the prediction contradicted the actual observations. Thus, the retinal generation notion is denied. More detailed deductions are given in the discussion.

In Experiment 2, the coherent percepts during the induction decreased as the inducing contrast increased. This result indicates that stronger eye-based grouping^23^,^24^,^25^ and (or) weaker pattern-based grouping^23^,^26^,^27^,^28^,^29^ correlate with stronger retinal and (or) cortical output signals. One would thus predict less coherent percepts in the FCI AIs if retinal signals determine grouping, because most likely the retinal signal is stronger in the FCI AIs than the AIM inductions. Alternatively, one may predict roughly an equal amount of predominance between the FCI AIs and AIM inductions if the summed cortical signal mainly determines grouping (Fig. 5 also assists in illustrating the deductions). However, we found much more coherent percepts for the FCI AIs than for the AIM inducers. This suggests that AI formation should also involve cortical mechanisms, and that there are likely multiple sources for AI signals in the visual system. Presumably, these cortical sources for AI signals might derive from rebound responses of cortical neurons after adaptation, and is an open question that may be tested in future work.

So what caused the increased interocular grouping in the AIs? According to the hybrid model of binocular rivalry^23^, coherent percepts should be caused by pattern-based grouping^26^,^27^,^28^,^29^, but disturbed by eye-based grouping that facilitates the perception of monocular patterns^24^,^25^. During the AIM induction, the retina is the unique source of the input signals. However, for the FCI AIs, the input signals (*i.e*. AI signals) are generated from multiple sites along the visual pathway. Many of these cortical signal origins may be binocular neurons representing the pattern-based grouping. Thus the probability of pattern-based grouping may be higher in the FCI AI condition than in the AIM induction condition, leading to more coherent percepts in the former.

There have been reports of differences in adaptation properties between monocular and binocular cells, suggesting substantially less adaptation in binocular cells over monocular cells^30^,^31^,^32^. One possibility then is that the high contrast stimuli of the present study particularly reduced monocular gain, but left binocular cells rather unaffected. During the afterimages, monocular cells may have adapted while binocular cells were spared and still responded vigorously. One caveat with this line of reasoning is that binocular cells receive their inputs from monocular cells^33^,^34^, and that their responses may correspond to the mean of the monocular cells^30^. As monocular cells adapt and reduce their gain, binocular cells will receive correspondingly weaker signals and in turn become less excited. Therefore, a slightly more complicated arrangement of interaction between monocular and binocular cells is required to account for the present findings. Certainly, further investigation is required to resolve the circuitry underlying the perceptual result reported in this study.

It should be noted that a previous study by Klink and colleagues found that over prolonged exposure to binocular rivalry images, the perception of mixtures of the two monocular images became more prevalent^35^. Broadly speaking, both this and our work suggest that adaptation to binocular rivalry images can lead to increased binocular integration. However, their findings differ from ours in a few important aspects. Firstly, the two studies are believed to reflect different kinds of binocular integration. The mixed percepts in Klink *et al*.’s^35^ work are either piecemeal mixtures or superimpositions of the monocular patterns. Piecemeal perceptions were usually composed of an uncertain amount of randomly distributed patchwork-like zones of local monocular dominance in their study. The stable percepts in our work could be systematically categorized into four fixed regular types (see Fig. 1). The formation of these four types of percepts should be subject to the mechanisms of perceptual grouping, obeying the Gestalt theory of perception^36^, e.g. collinearity^37^. Secondly, it is very unlikely that Klink *et al*.’s^35^ effect accounts for our results. The accumulation of Klink *et al*.’s^35^ effect strongly relies on the constant eye-of-origin information of the rival images. However, the eye-of-origin information of the rival patterns in our study was randomized across the trials. Also, the adaptation duration in each trial was only 55 s, while in Klink *et al*.’s^35^ work it was 35 min. Thirdly, the main finding in the current work is about the enhanced interocular grouping in the AIs relative to the inducers. However, Klink *et al*. did not investigate AIs^35^, thus their work provides no information about AIs.

The present study supports the view that cortex is directly involved in the generation of AI signals by showing distinct interocular grouping for AIs and physical stimuli of similar appearance. Binocular rivalry is considered to occur at multiple stages of visual processing^26^,^38^,^39^,^40^,^41^,^42^,^43^. The observed phenomenon suggests that perceptual alternations in binocular rivalry may depend on both the early feed-forward signals and later controlling mechanisms that favor pattern coherency such as binocular neurons.

## Methods

In all the experiments of the present study, all participants had normal or corrected-to-normal vision. Experimental procedures were approved by the Institutional Review Board of the Institute of Psychology, Chinese Academy of Sciences, and all methods were performed in accordance with the relevant guidelines and regulations. Informed consents were obtained from all the subjects in all the experiments of the present study.

### Experiment 1

#### Subjects

10 naïve subjects (6 males, ages ranging from 21 to 26 years) participated in this experiment.

#### Apparatus

Stimuli were presented on a gamma-corrected 27-inch Asus VG278HE LED monitor (1920 pixel × 1080 pixel resolution at the refresh rate of 120 Hz) in a dark room. Subjects viewed the display at a distance of 100 cm through a pair of shutter goggles (Nvidia 3D vision wireless glasses) that alternated between left and right eye occlusion with each frame. A chin-rest was used to minimize head motion. The display was calibrated with a Photo Research PR-655 spectrophotometer. To calibrate the display, we measured the luminance gamma curves and inverted them using a look-up table. The mean luminance of the screen was 57.4 cd/m^2^.

#### Stimuli

Stimuli in each eye contained a centrally presented 2 × 2 square array of sinusoidal gratings (full contrast, 1 cyc/deg), with each grating subtending 3° (see Fig. 1). A black-and-white frame (7° × 7°, width: 0.09°) surrounded each square array to aid fusion. Presented to one eye, the elements in two diagonal quadrants were vertical gratings, while those in the other quadrants were horizontal. The orientations of the corresponding elements in the other eye were orthogonal, thus binocular rivalry could happen at each element position. Theoretically, complete interocular grouping might produce a coherent percept of an all-vertical or all-horizontal grating. With incomplete grouping, subjects may perceive two types of partially integrated patterns to avoid having them see the monocular grating arrays. The four possible types of percepts are listed in the lower right part of Fig. 1. The stimuli remained constant within each trial, with the spatial phases reversed across trials. The stimulus pattern and its eye of origin were randomized across trials. All stimuli were presented foveally on a mid-gray background with a red bull’s eye central fixation point (0.36° in diameter).

#### Procedure

At the beginning of each trial, dynamic white noise (6° × 6°) was presented for 3 s to minimize any residual afterimage from the previous trial. The induction in each trial lasted for 55 s. During both the induction and AI phases, subjects were told to maintain fixation and report which of the four types of patterns they saw by pressing and holding one of the four arrow keys. They were also told to release all the keys for the last 2 s in the induction phases following a beep cue, in case the final response in the induction phases conflicted with the initial response in the AI phases. During both the induction and AI phases, subjects sometimes experienced perceptual alternations of patterns defined as the same type in Fig. 1. For example, it was common for them to see rivalrous all-vertical and all-horizontal AIs. However, we did not assign additional keys to distinguish between these within-type perceptual alternations as it could have introduced confusion, and the focus of the study did not warrant that level of precision in the data. Once the induction ceased, subjects were presented with a mean field excepting the fusion frames and fixation point which remained on the screen. AIs have been found to fluctuate in visibility before they finally cease to be visible^44^,^45^. To acquire sufficient data during the AI phases, we instructed subjects to close their eyes for 2 s and then reopen the eyes whenever the AIs intermittently disappeared. This would allow the AIs to reappear, though they would be fainter each time they reappeared. Subjects were told to stop a trial by pressing the spacebar when they had closed and reopened the eyes for three times but still could not make the AIs reappear. After a brief practice period explained below, each subject completed 15 trials.

Using four arbitrary keys to indicate percept type is potentially confusing, and incorrect use of the keys naturally disqualifies the results. To reduce the risk of percept-to-key mapping errors, all subjects were required to practice the task in two successive stages. In the first stage of practice, binocularly congruent grating patterns were presented to them, which simulated all the possible percepts of the four categories. This stage terminated once the subjects had made no incorrect responses over 6 continuous minutes. Thereafter the second stage started, in which they viewed dichoptic stimuli as in the formal experiment. Taken together, the practice would substantially have limited the risk of incorrect response mapping and ensured that our subjects fully understood and used the four categories to complete the task.

### Experiment 2a

#### Subjects

Twenty subjects (7 males, ages ranging from 19 to 34 years) participated in Experiment 2a. Two of them had participated in Experiment 1. Nineteen of them were naive to the experimental hypotheses.

#### Stimuli and procedure

Experimental parameters were the same as in Experiment 1 except for the following changes. Stimuli were presented on a gamma-corrected 22-inch Dell P1230 CRT monitor (1024 pixel × 768 pixel resolution at 85 Hz), which was driven by a Bits# 14-bit video card (Cambridge Research Systems). Stimuli were viewed at a distance of 88.5 cm in a dark room through a mirror stereoscope. The mean screen luminance was 51.9 cd/m^2^. The fusion frames were removed during the AI phases as they might reduce the visibility of AIs especially when the inducing contrast was low.

In a separate preliminary test, one author and one naïve subject empirically measured the apparent contrast of FCI AIs. See the Results section for more details.

In each block of the formal experiment, subjects completed 24 trials for each of the test contrasts. In total, 10 blocks (120 trials) were completed, with the sequence of the five testing contrasts counterbalanced across subjects using Latin square series.

### Experiment 2b

#### Subjects

Seventeen subjects were recruited (8 males, ages ranging from 18 to 29 years). One of them was excluded because he could only see the lowest contrast inducer 14.3% of the induction duration, whereas the other subjects saw the lowest contrast inducer 86.1% ± 14.5% of the inducer duration. All subjects were naive to the experimental hypotheses.

#### Stimuli and procedure

The details of this experiment were the same as Experiment 2a except for the following. The full contrast inducer was identical to that of Experiment 2a. However, the lower contrast inducing grating arrays were filtered with a modified 3rd-order Butterworth filter (cutoff at 1 cyc/deg), to retain the energy for spatial frequencies lower than 0.5 cyc/deg. The reason for using the filter was to reduce the energy of higher frequency components as much as possible while avoiding severe distortions of the image (see Supplementary Fig. S1). To revive AIs, instead of requesting blinks, a 1-s display containing a black screen was adopted. This reduced the unnecessary blinks during the AI phases. If no responses were made within 3 s after a black screen, another black screen would be presented. If the AIs still would not reappear after 3 successive black screens, subjects ended the current trial by pressing a specified key.

Because the authors sometimes perceived plaids while piloting the test, and since that perception did not fit into the four perceptual categories used in the previous experiments, subjects in this experiment were assigned an extra response key for reporting plaids. Type V represents the perception of a plaid. Besides, if the AI at any quadrant(s) disappeared, subjects were instructed to release all the keys.

As in Experiment 2a, subjects completed a separate preliminary test to measure the apparent contrast of FCI AIs. In contrast to Experiment 2a, this test was now taken by each subject. The test involved a total of 5 trials. The contrast for the point of subjective equality ranged between 0.02 ± 0.01 and 0.42 ± 0.16 (for details, see Supplementary Table S2). Here 1 represented the full contrast. Three levels of the mimicking contrast were individually selected for each subject: the low and high contrasts were the lowest (0.02 ± 0.01) and highest perceived contrasts (0.42 ± 0.16) during the AI phases. Furthermore, a medium contrast was computed by taking the square root of the product of the lowest and highest contrasts which yielded an average of 0.10 ± 0.02.

### Experiment 2c

#### Subjects

Twelve subjects (8 males, with an age range from 20 to 25 years) participated in Experiment 2c. All of them were naive to the experimental hypotheses.

#### Stimuli and procedure

Each subject completed two sessions. In one session, the stimuli and procedure were identical to those of Experiment 1. Since each grating element contained three full sinusoidal cycles, there was a unified spatial phase everywhere on the coherent pattern under perfect interocular grouping. Subjects could also perceive a coherent AI pattern with a single spatial phase. We term this induction condition the “in-phase” session.

In the other session, the grating elements of two diagonal quadrants in one eye were out of phase with those of the two opposite diagonal quadrants in the other eye (See Fig. 3a). When coherent AI patterns were perceived, this stimulus arrangement lead to perception of patterns in which the spatial phase in each quadrant was reversed with respect to those of the adjacent quadrants. We call this condition the “out-of-phase” session.

### Experiment 3

#### Subjects

One author (M.B.) and 13 naive subjects (7 males, ages ranging from 20 to 36 years) participated in this experiment. One of them also participated in Experiment 2b. All subjects had normal or corrected to normal vision. Two subjects’ data sets were removed from further analysis because the proportions of valid eye movement data were less than 85%.

#### Stimuli and procedure

Experimental parameters were the same as in Experiment 2b except for the following changes. The AI-mimicking inducers with 0.04 contrast were tested in this experiment, and the grating arrays were also low-pass filtered as in Experiment 2b. In total, subjects completed six blocks, each block containing five trials. Gaze calibration was performed at the beginning of each block. Throughout a block, subjects were required to keep their heads still on the head and chin-rest. A short rest period was given after the end of each block. Subjects also completed 15 extra trials with full contrast induction as in Experiment 1 in a separate session with eye tracking. The behavioral results confirmed that the basic effects existed in these subjects (the predominance for the coherent precepts was higher for the AIs (54.4%) than for the inducers (19.8%), *t*(11) = 5.10, *p* = 0.00034, *d* = 1.48, 95% CI [0.20, 0.49]).

#### Eye movement recording

Eye movements for both eyes were recorded using a fast video-based eye movement monitor (SMI RED250, temporal resolution 250 Hz, instrument noise 0.03 deg RMS) in its off-the-shelf configuration. The eye tracker was positioned between the CRT monitor and stereoscope. The stereoscope was installed with cold mirrors that were transparent to infrared light, allowing for tracking eye movements through the stereoscope. Gaze calibration was performed using the conventional nine-point calibration routine.

#### Eye movement analyses

##### Blink

When blinks occur, the eye-tracker will lose track of the pupil information, which usually produces missing samples during the recording. However, errors in data acquisition may also cause missing samples. To differentiate blinks from acquisition errors, we adopted two criteria for blink detection. Firstly, the minimum duration of missing samples caused by blinks should be no shorter than 12 ms. Secondly, the interval between any two successive detected blinks must be no shorter than 300 ms (see Supplementary Fig. S3b). With these criteria, the instant and frequent missing samples due to acquisition errors could be distinguished from blinks and were thus removed (frequency for missing samples due to acquisition errors amounted to 0.066% ± 0.069% of the full contrast induction period and 0.076% ± 0.114% of the AI-mimicking induction period. As shown in Supplementary Fig. S4, most missing samples of this type occurred for a single or two continuous samples, *i.e*. 4 or 8 ms in duration).

##### Saccades and microsaccades

When identifying saccades or microsaccades, we first rejected the portions of data in which blinks or semi-blinks occurred, as well as data sampled 200 ms before and after blinks^46^. Data with very fast decreases or increases of pupil area (>7.90 mm^2^/sample or 50 pixel^2^/sample) were deemed as semi-blinks where the pupils were never fully occluded^46^. A 4^th^-order Savitzky-Golay filter with a 156 ms window (39 data points) was then used to reduce the noise^47^.

After these preprocesses, microsaccades and saccades were automatically detected by using a velocity-based algorithm^48^. First, the velocity for each fixation position was calculated based on the time series of fixation positions, which produced a distribution in the 2D velocity space (see Supplementary Fig. S3d). Second, saccades were defined when the velocity was more than four times the median-based SD of the velocity distribution (λ = 4) and when its duration was larger than 24 ms. In addition, to avoid defining potential overshoot corrections as new saccades, only intersaccadic intervals longer than 20 ms were retained for analysis^49^. Finally, saccades less than 1° in magnitude were considered to be microsaccades (see Supplementary Fig. S3c).

##### Drift

Involuntary drifts of the eyes were defined as motion in the remaining periods excluding saccades, microsaccades, and blinks^50^. Periods of semi-blinks were also excluded. The lengths of drifts were calculated using the sum of the eye position shifts during all the drifting periods. Bear in mind that drift lengths thus assessed are also influenced by tremors and instrument noise.

For each eye, we analyzed the number of blinks, saccades, microsaccades, and lengths of drifts in each trial. For each subject, these indices were averaged across the two eyes. Thereafter, we conducted correlation analyses between the predominance of coherent percepts and each eye movement index. After averaging the eye movement indices across trials for each subject, we also conducted similar correlation analyses across the subjects. A Pearson product-moment correlation (*r*) analysis was performed if the distributions of the original or transformed data were normal (square root-transformation for the number of blinks, microsaccades, and saccades; logarithmic transformation for the drift length and predominance of type IV percept). Otherwise, a Spearman’s rank correlation (*r*_*s*_) was computed.

## Additional Information

**How to cite this article**: Dong, B. *et al*. Cortical mechanisms for afterimage formation: evidence from interocular grouping. *Sci. Rep.* **7**, 41101; doi: 10.1038/srep41101 (2017).

**Publisher's note:** Springer Nature remains neutral with regard to jurisdictional claims in published maps and institutional affiliations.

## Supplementary Material

Supplementary Information

## Figures and Tables

**Table 1:** Predominance for every inducing contrast and the FCI AI in Experiment 2a and Experiment 2b.

	Contrast	Perceptual type (*M* ± *SD*)				
		I	II	III	IV	V
Exp. 2a	0.04 (AIM)	19.9% ± 14.4%	15.3% ± 9.0%	26.2% ± 9.0%	38.6% ± 14.1%	
	0.082 (AIM)	17.5% ± 10.5%	17.5% ± 7.8%	30.4% ± 8.8%	34.6% ± 11.8%	
	0.17 (AIM)	18.9% ± 10.3%	19.2% ± 7.0%	33.7% ± 9.3%	28.2% ± 9.2%	
	0.35 (AIM)	22.1% ± 13.6%	19.3% ± 8.0%	32.9% ± 10.2%	25.7% ± 8.4%	
	Full (AIM)	17.4% ± 10.7%	20.1% ± 8.3%	34.3% ± 10.2%	28.2% ± 10.7%	
	Full (AI)	4.7% ± 5.5%	10.0% ± 9.3%	14.7% ± 11.0%	70.6% ± 22.3%	
Exp. 2b	Low (AIM)	7.6% ± 6.8%	15.6% ± 7.8%	21.9% ± 10.5%	41.0% ± 13.1%	0.05% ± 0.20%
	Med.(AIM)	11.1% ± 7.1%	21.1% ± 8.0%	28.4% ± 7.6%	35.8% ± 11.2%	0.09% ± 0.23%
	High (AIM)	11.1% ± 8.0%	23.0% ± 6.1%	30.8% ± 7.6%	30.9% ± 10.5%	0.04% ± 0.10%
	Full (AIM)	20.5% ± 11.0%	22.8% ± 7.5%	31.6% ± 11.7%	21.7% ± 8.2%	0.002% ± 0.01%
	Full (AI)	5.5% ± 7.1%	15.9% ± 11.7%	18.2% ± 12.0%	58.2% ± 23.6%	2.4% ± 6.2%

**Table 2:** Correlation between the predominance of coherent percepts and eye movement indices.

Subject	Number of blinks		Number of saccades		Number of microsaccades		Drift length	
	*r*/*r*_*s*_	*p*	*r*/*r*_*s*_	*p*	*r*/*r*_*s*_	*p*	*r*/*r*_*s*_	*p*
1	**−0**.**4901**	**0**.0060*	<0.0001	1	0.2834	0.1291	*0.0638*	*0.7368*
2	*0.1825*	*0.3343*	0.0474	0.8036	−0.1875	0.3210	−0.0896	0.6378
3	−0.0240	0.8998	0.3203	0.0844	0.0845	0.6569	0.1192	0.5304
4	***0**.**5756***	**0**.0009*	*−0.1883*	*0.3189*	*−0.2430*	*0.1957*	*0.0843*	*0.6567*
5	−0.0634	0.7393	−0.0506	0.7906	−0.0833	0.6615	*0.1711*	*0.3645*
6	0.2145	0.2550	0.1019	0.5921	**0**.**3839**	**0**.0362*	−0.1776	0.3477
7	*0.0732*	*0.7005*	0.1105	0.5611	0.0092	0.9613	*0.1017*	*0.5916*
8	0.1849	0.3280	−0.2338	0.2136	−0.1647	0.3846	0.3359	0.0695
9	−0.0974	0.6086	−0.2430	0.1957	−0.1561	0.4099	0.0392	0.8372
10	0.0855	0.6533	−0.1680	0.3750	−0.1610	0.3955	−0.0085	0.9645
11	−0.0957	0.6148	−0.2046	0.2782	0.3381	0.0677	−0.1881	0.3195
12	−0.0289	0.8795	−0.2567	0.1708	−0.0772	0.6851	−0.2980	0.1098

We performed Pearson product-moment correlation analyses if the original or square-rooted/logarithmic data were normally distributed. Otherwise, we computed Spearman’s rank correlation analyses (see those shown in italic). Significant correlations are shown in bold.
