# Supplementary material for: Cortical mechanisms for afterimage formation: evidence from interocular grouping
Source: Sci Rep. 2017 Jan 23;7:41101. doi: 10.1038/srep41101 (PMC5253736; doi:10.1038/srep41101)
Supplement: Supplementary Information [file srep41101-s1.pdf]

Cortical Mechanisms for Afterimage Formation: Evidence from Interocular Grouping

Bo Dong[1,3], **Linus Holm**[2], **Min Bao**[1,*]

[1] CAS Key Laboratory of Behavioral Science, Institute of Psychology, Beijing 100101, P.R. China
[2]Department of Psychology, Umeå University, S-901 87 Umeå, Sweden
[3]University of Chinese Academy of Sciences, Beijing 100101, P.R. China

[*]Corresponding. baom@psych.ac.cn

Supplemental introduction on "indirect proof"

In case some readers are not familiar with the "indirect proof", here we show a simple example of the "indirect proof" in algebra.

Given that

$a^3 + b^3 = 2,$ ………………………………………………………………….... (1)

try demonstrating that

$a + b \leq 2.$ ……………………………………………………………...… (2)

Below is the deduction process of the "indirect proof".

Assuming that

$a + b > 2,$ …………………………………………………………….…... (3)

we can get a deduction

$a > 2 – b.$ …………………………………………………………….…... (4)

According to a fact that can be easily demonstrated from the properties of inequalities, we further know that

$a^3 > (2 - b)^3$ ..……………………………………………………………….. (5)

thus

$a^3 > 8 - 12b + 6b^2 – b^3,$

$a^3 + b^3 > 6b^2 – 12b + 8 = 6(b-1)^2 +2.$

Since $6(b-1)^2 +2 \geq 2$, we reach a prediction that

$a^3 + b^3 > 2.$. ……………………………………………………………….. (6)

However, the prediction (6) contradicts the already-known fact (1). Accordingly, the opposite hypothesis (3) is denied. In this math example, we already know a fact (1) and want to demonstrate a hypothesis (2). Using the indirect proof, we can first assume that the opposite hypothesis (3) is true. We then derive a deduction (4) from the opposite hypothesis (3). By combining the deduction (4) with another fact (5), we can further reach a prediction (6). Since the prediction (6) contradicts the already-known fact (1), the opposite hypothesis (3) has to be denied. As a result, the hypothesis (2) is validated.

As we note, the present study tries to prove the cortical generation notion (2). Thus, we would first assume that the retinal generation notion (3) is true. Generally, AIs appear after adaptation to a stimulus, and adaptation reduces the gain of the visual system. That is, the visual system is not in the same state during AIs as it is during the presentation of a stimulus with AI-similar contrast. Assuming that the retinal generation notion is correct, i.e. the retina is the only origin of AI signals, comparable contrast appearance would require stronger retinal signals in the AIs than in the inductions with AI-similar contrast (4). According to the findings in Experiment 2 that increased inducing contrast caused decreased interocular grouping during the inductions (5), we may reach a

prediction (6) that more frequent monocular patterns should be perceived in the AIs than in the inductions with AI-similar contrast. However, this prediction (6) contradicts the empirical observations (1) of subjects actually seeing the fully coherent stripes significantly more often in AIs. As a result, the retinal generation notion (3) is denied. The present study thus suggests that AI formation should also involve cortical processes in addition to the retinal mechanisms (2).

Supplemental figures and tables

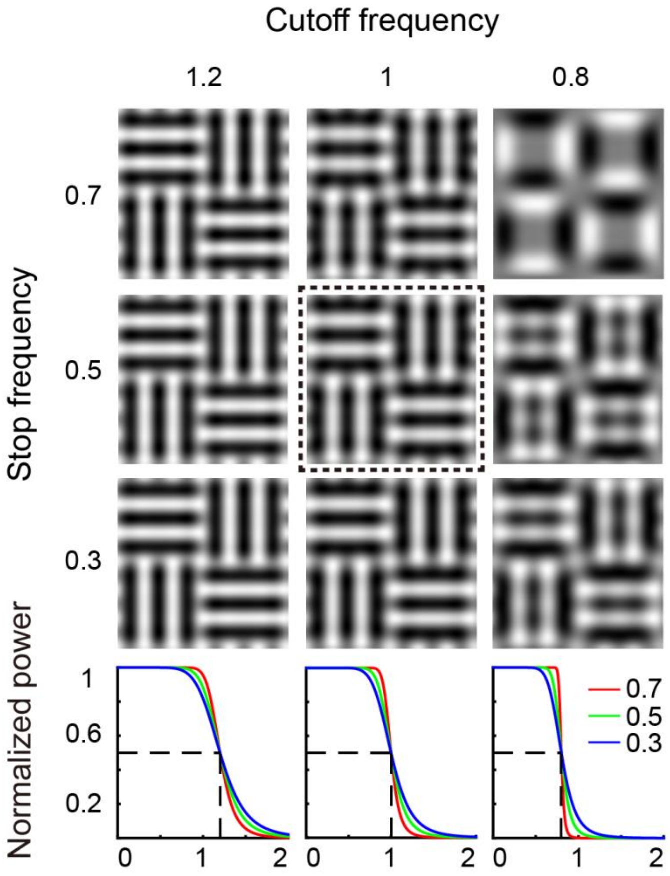

Figure S1. Candidates for the Butterworth filters and the filtered stimuli. Images were distorted severely for cutoff frequencies lower than 1 cpd. No obvious distortion was found when the stop frequency was between 0.3 cpd to 0.7 cpd with a higher cutoff frequency (≥ 1 cpd). The central image (framed with dotted lines) was the one selected in the study. The cutoff frequency was 1 cyc/deg. The filtering retained the energy for spatial frequencies lower than 0.5 cyc/deg.

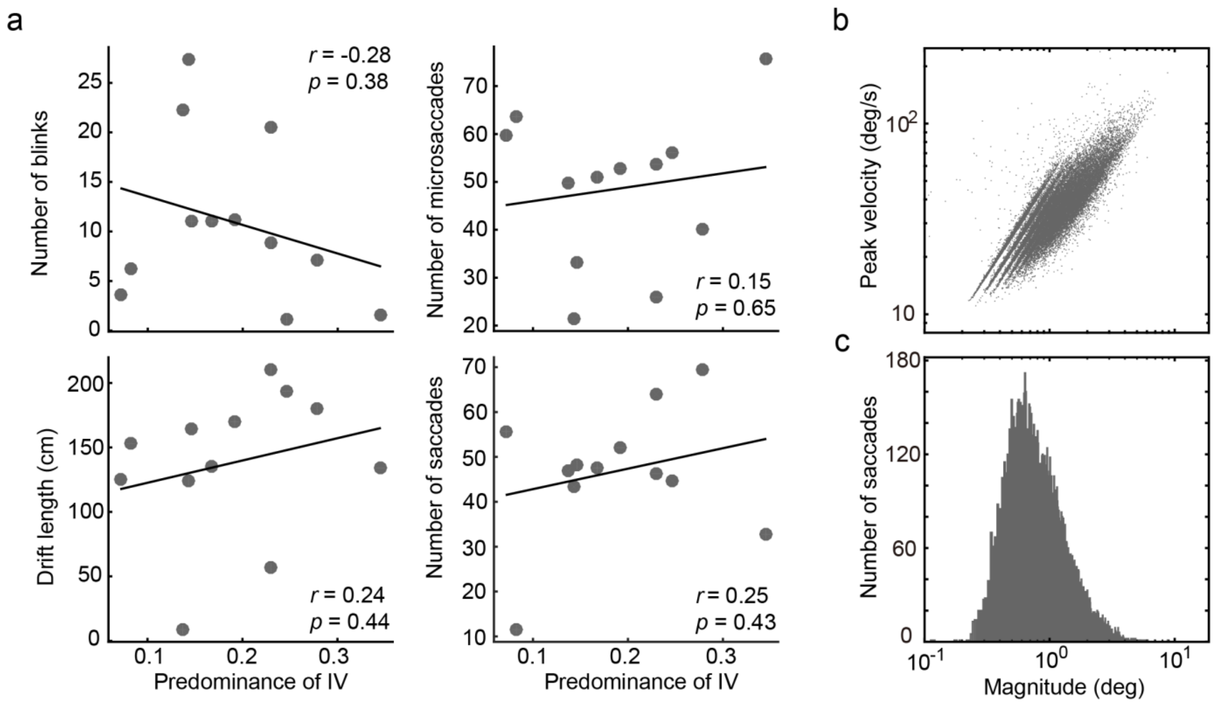

Figure S2. Results of eye movement during the period of full contrast induction in Experiment 3: **(a)** Correlations between the perceived predominance of type IV percepts and the number of blinks (upper left), microsaccades (upper right), saccades (lower right) , and drift length (lower left) across the subjects. Each dot represented a subject. No significant correlation was observed between the rate of interocular grouping during induction and any of our eye movement indices (number of blinks, M = 10.97, SD = 8.36, $r(10)$ = - 0.28, p = 0.38; saccades, M = 46.89, SD = 14.65, $r(10)$ = 0.25, p = 0.43; microsaccades, M = 48.58, SD = 15.75, $r(10)$ = 0.15, p = 0.65; and length of drifts, M = 138.00 cm, SD = 56.80 cm, $r(10)$ = 0.24, p = 0.44). **(b)** Microsaccadic and saccadic peak velocity–magnitude relationship for all subjects combined. Each dot represents a microsaccade or a saccade with peak velocity indicated on the *y*-axis and magnitude indicated on the *x*-axis. **(c)** Magnitude distribution of microsaccades and saccades.

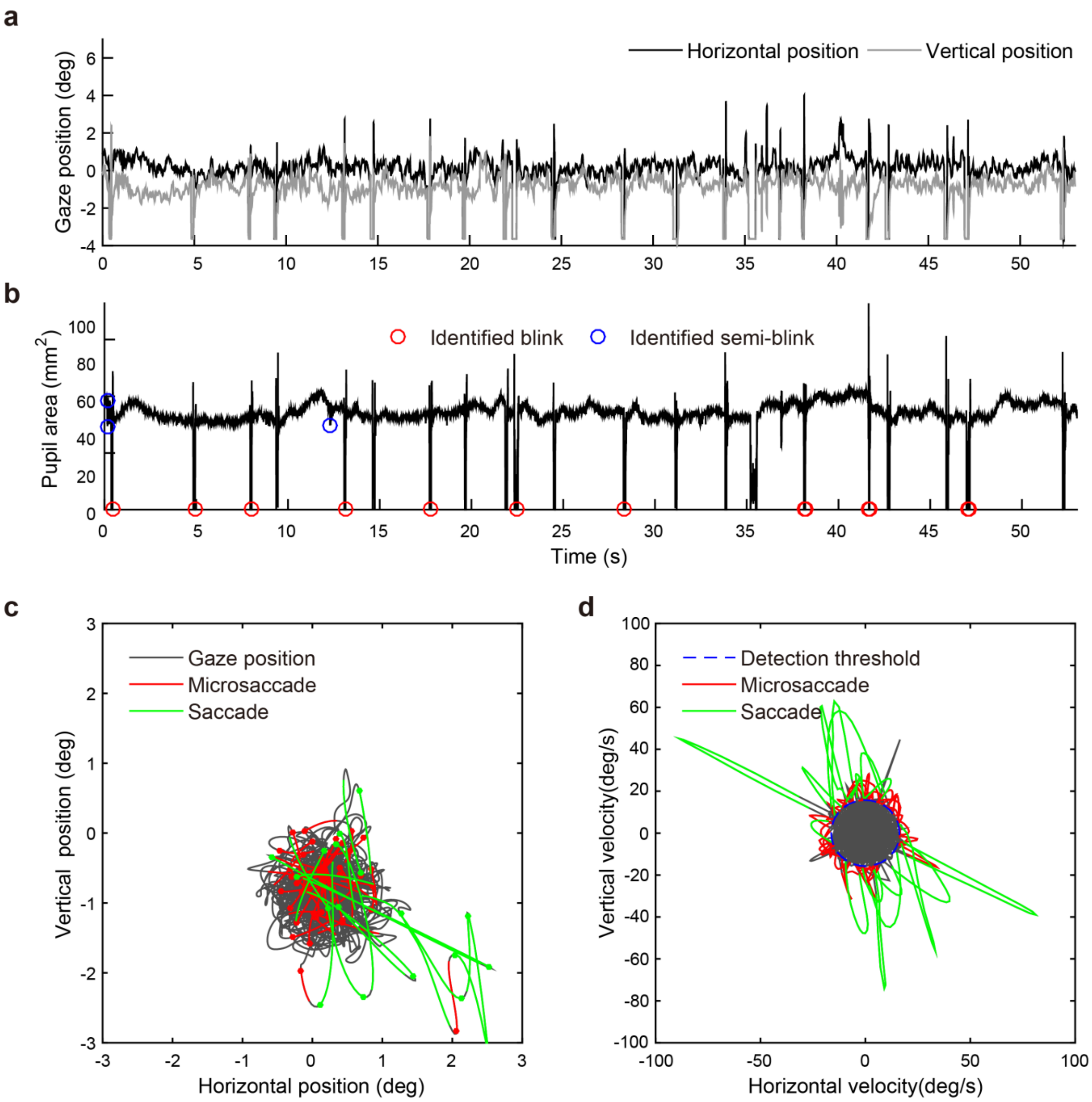

Figure S3. Fixational eye movements and detection of blinks, microsaccades and saccades, recorded from a subject's left eye. **(a)** Time course of the gaze positions. Horizontal (black lines) and vertical (gray lines) coordinates of the gaze positions were shown separately. **(b)** Time course of the pupil areas. Blinks (red circles) and semi-blinks (blue circles) were identified by our algorithm (red lines). **(c)** Plots of the gaze positions, microsaccades and saccades. Gaze positions are indicated by black lines. Microsaccades are small (< 1deg) but rapid events which can be identified by their approximately linear appearances (red lines) and saccades are the larger ones (> 1deg, green lines). **(d)** A plot of the trajectory in 2D velocity space shows considerably higher peak velocities for microsaccades (red lines) and saccades (green lines) compared to other components of eye movements. Detection thresholds (blue dot circle) were computed separately for horizontal and vertical components.

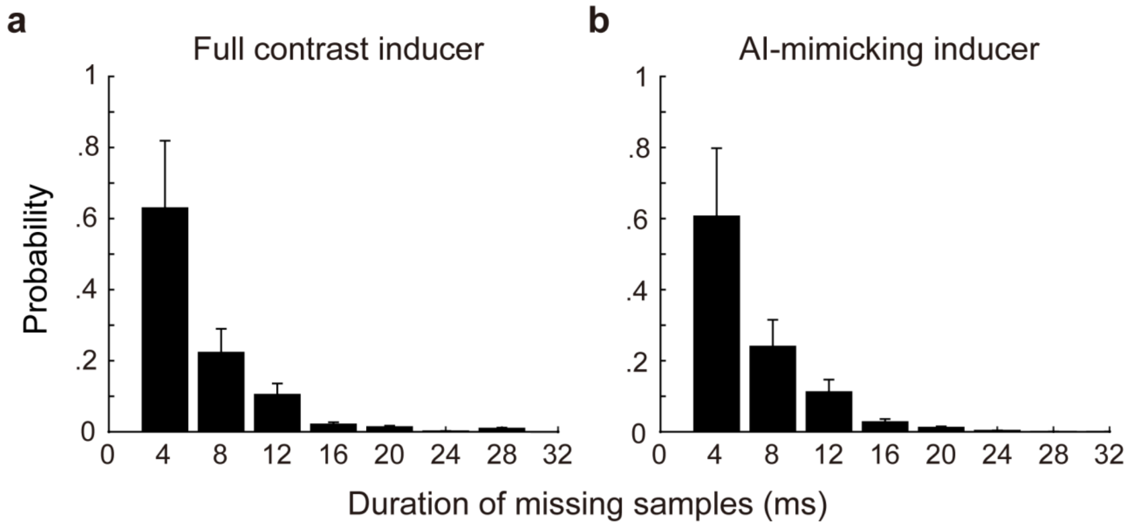

Figure S4. Frequency of missing samples due to eye movement acquisition errors was 0.066% (*SD* = 0.069%) for full contrast inductions and 0.076% (*SD* = 0.114%) for AI-mimicking inductions. As shown in this figure, most missing samples due to acquisition errors occurred for single or two continuous samples, *i.e.* 4 or 8 ms in duration. The probability is computed based on the relative frequency of missing samples. Error bars represent standard errors of the means.

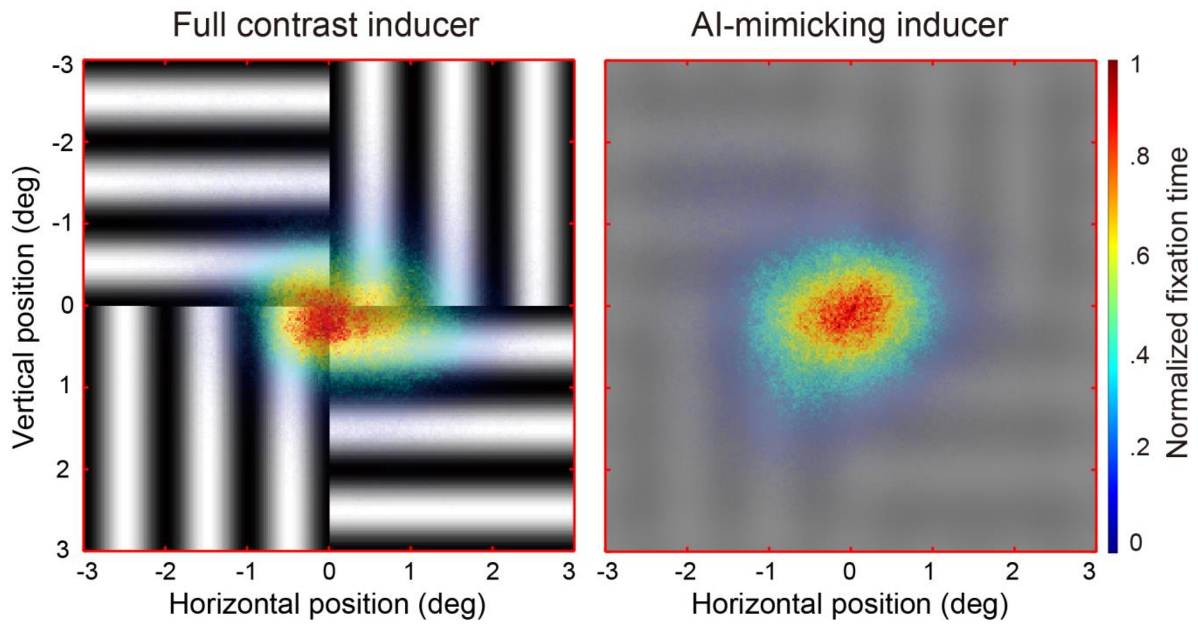

Figure S5. The spatial distributions of gaze positions across all the subjects during the full contrast (left) and the AI-mimicking (right) inductions. The distribution results showed that most subjects in Experiment 3 maintained steady central fixations.

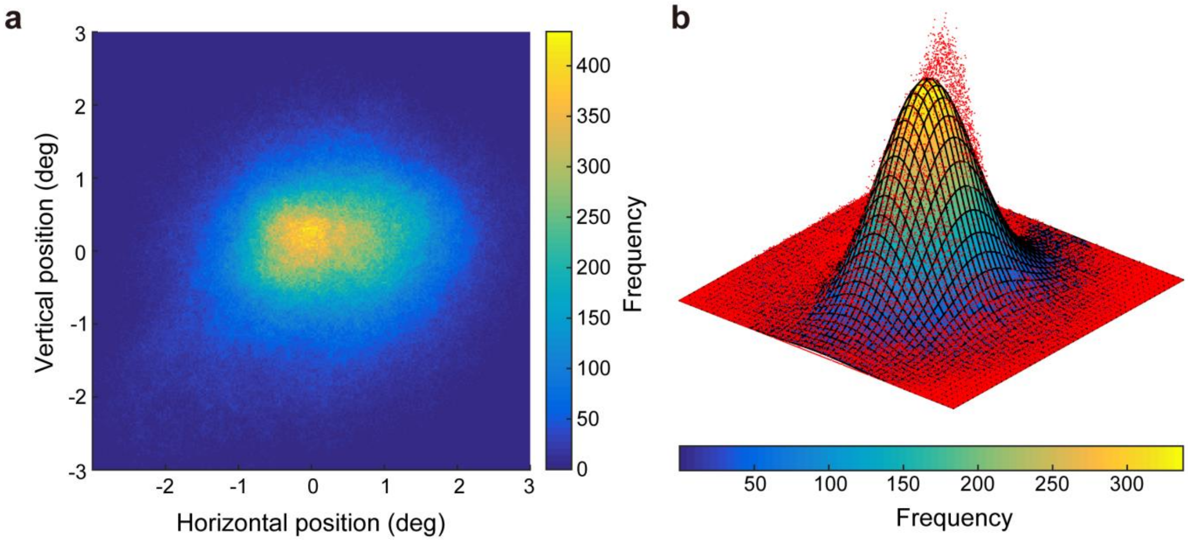

Figure S6. The 2D Gaussian model (curved surface in **b**) fitted to the spatial distributions of the gaze positions (**a**, and red dots in **b**) for all subjects during full contrast induction phase. The equation has four free parameters:

$$f(x,y) = Ae^{-\left[\frac{(x-x_0)^2}{2\sigma_x^2}+\frac{(y-y_0)^2}{2\sigma_y^2}\right]}$$

A is the height of the amplitude, x_0, y_0 are the centers, σ_x, σ_y are horizontal and vertical standard deviations. Results about the fitting are in Table S3.

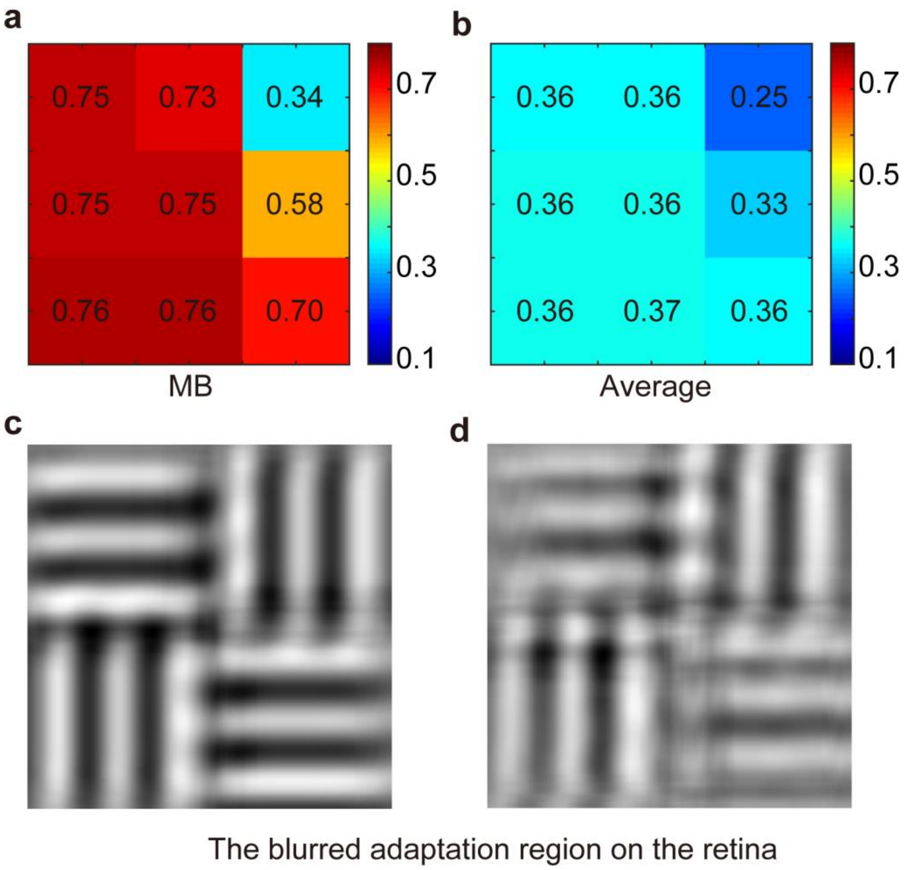

Figure S7. In Experiment 2b, we selected the middle patch in Figure S1 based on our empirical feeling for the AIs. After acquiring the eye movement data, we re-examined whether our selection was appropriate. The results were shown in this figure. For each trial (55 s) of each subject in Experiment 3, we estimated the image on the retina at each time point (i.e. 4 ms for the 250 Hz sampling rate of the eye movement recording) based on the x y coordinates of the eye fixations. These images were superimposed on each other to simulate the blurred adaptation region on the retina. Note that this simulation rests on a simple model where we do not consider the time constant of decay on the retina, because the time constant of adaptation is unknown and may vary across stages of the visual hierarchy. We then performed a pixel-by-pixel correlation analysis between this simulated image and each of the 9 possible candidates shown in Figure S1. Specifically, each image array was reshaped into an N by 1 vector. Then we ran a Pearson's correlation analysis between each pair of vectors to obtain a 3-by-3 array of the correlation coefficients for the 9 candidates. For each subject, the arrays for the 15 trials were averaged to show the average correlation coefficients for the 9 candidate patches. **(a)** The correlation coefficient for each filtered patch (arranged in the manner as in the 9 candidates in Figure S1) in a typical trial for the experienced subject MB. **(b)** The grand average coefficients of the correlation for all subjects. **(c)** The simulated image for one trial of the subject MB. **(d)** The simulated image for one trial of a naïve subject. Subject MB is very experienced in psychophysical experiments, therefore he can keep better central fixation than other naïve subjects. This may explain why the correlation coefficients for his data were much higher than those of the grand average data.

Trial	The contrast of the FCI AIs			
	JB		BD	
	Maximum	Minimum	Maximum	Minimum
1	0.64	0.04	> 0.64	0.04
2	0.64	0.04	0.64	0.04
3	0.64	0.04	0.64	0.08
4	0.64	0.04	0.32	0.04
5	0.64	0.04	0.32	0.04

Table S1. The apparent contrast of the FCI AIs in a preliminary test of Experiment 2a.

Sub.	Trial 1		Trial 2		Trial 3		Trial 4		Trial 5		**Mean**		
	Max	Min	Max	Min	Max	Min	Max	Min	Max	Min	**Max**	**Med**	**Min**
1	0.08	0.02	>0.64	0.08	>0.64	0.04	0.64	0.02	>0.64	0.02	**0.53**	**0.14**	**0.04**
2	0.32	0.02	0.32	0.02	0.64	0.02	0.64	0.02	0.32	0.02	**0.45**	**0.09**	**0.02**
3	0.16	0.04	0.08	0.02	0.64	0.04	0.32	0.04	0.16	0.04	**0.27**	**0.10**	**0.04**
4	0.32	0.08	0.32	0.02	0.32	0.04	0.64	0.04	0.64	0.04	**0.45**	**0.14**	**0.04**
5	0.16	0.04	0.08	0.02	0.32	0.04	0.32	0.02	0.32	0.04	**0.24**	**0.09**	**0.03**
6	0.16	0.02	0.08	0.02	0.64	0.02	0.32	0.02	0.16	0.02	**0.27**	**0.07**	**0.02**
7	0.32	0.02	0.16	0.02	0.64	0.02	0.64	0.02	0.32	0.02	**0.42**	**0.09**	**0.02**
8	0.64	0.02	0.64	0.02	0.64	0.02	0.64	0.02	0.64	0.02	**0.64**	**0.11**	**0.02**
9	0.32	0.04	0.64	0.02	0.32	0.02	0.64	0.02	0.64	0.02	**0.51**	**0.10**	**0.02**
10	0.16	0.02	0.16	0.02	0.16	0.02	0.32	0.02	0.32	0.02	**0.45**	**0.09**	**0.02**
11	0.04	0.02	0.32	0.02	0.32	0.02	0.32	0.02	0.32	0.02	**0.27**	**0.07**	**0.02**
12	0.04	0.02	0.32	0.02	0.32	0.02	0.64	0.02	0.64	0.02	**0.40**	**0.09**	**0.02**
13	0.08	0.02	0.08	0.02	0.08	0.02	0.08	0.02	0.08	0.02	**0.08**	**0.04**	**0.02**
14	0.64	0.02	>0.64	0.02	0.64	0.02	>0.64	0.02	0.64	0.02	**0.64**	**0.11**	**0.02**
15	0.64	0.02	0.64	0.02	0.64	0.02	0.64	0.02	0.64	0.02	**0.64**	**0.11**	**0.02**
16	0.16	0.02	0.32	0.02	>0.64	0.02	>0.64	0.04	>0.64	0.04	**0.48**	**0.12**	**0.03**

Table S2. The apparent contrast of the FCI AIs in the preliminary test of Experiment 2b. Measured contrasts higher than 0.64 were taken as 0.64 when we calculated the mean.

Subject	Number of blinks		Number of saccades		Number of microsaccades		Drift length	
	r / r_s	p	r / r_s	p	r / r_s	p	r / r_s	p
1	-0.2930	0.2891	-0.3463	0.2061	0.1489	0.5963	0.0402	0.8870
2	*0.1160*	*0.6806*	0.3175	0.2489	-0.2527	0.3635	-0.0798	0.7773
3	0.2127	0.4466	-0.2562	0.3567	-0.2524	0.3641	0.3102	0.2605
4	-0.0887	0.7532	*-0.0287*	*0.9191*	-0.1845	0.5105	0.0772	0.7845
5	*-0.3745*	*0.1690*	0.4367	0.1036	**0.5400**	**0.0377***	-0.1959	0.4840
6	*-0.2457*	*0.3773*	*0.3214*	*0.2424*	***0.5559***	***0.0314****	*0.4643*	*0.0834*
7	-0.2435	0.3819	-0.0835	0.7672	0.2206	0.4294	0.0528	0.8517
8	-0.2647	0.3404	-0.1510	0.5912	0.0130	0.9633	0.3844	0.1571
9	-0.0229	0.9355	0.0690	0.8071	0.0588	0.8351	-0.0703	0.8035
10	0.1720	0.5400	**0.5970**	**0.0188***	0.1009	0.7206	-0.0830	0.7687
11	*0.2194*	*0.4320*	-0.2397	0.3895	0.0326	0.9081	0.0016	0.9956
12	*-0.0915*	*0.7458*	*0.2021*	*0.4700*	*0.1039*	*0.7124*	*0.0929*	*0.7435*

Table S3. Correlation between the predominance of coherent percepts and eye movement indices during full contrast inductions. We performed Pearson product-moment correlation analyses if the original or square-rooted /logarithmic data were normally distributed. Otherwise, we conducted Spearman's rank correlation analyses (see those shown in italic). Correlation coefficients for individual participants were shown in this table. None of the subjects displayed any significant negative correlation between the coherent percepts and any of the eye movement indices. Furthermore, three subjects displayed individually positive correlations between number of saccades / microsaccades and interocular grouping rate (shown in bold), which disagreed with the argument that fixation jitter during induction caused stronger interocular grouping in AIs. Therefore, the prevalence of coherent percepts does not appear to have been influenced by eye movements during full contrast inductions.

Subjects	A	x_0	y_0	σ_x	σ_y
1	104.2728	-0.2279	0.1694	0.4381	0.4148
2	69.0356	1.16536	0.4713	0.6224	0.4347
3	36.5907	-0.0320	0.0518	0.7204	0.5818
4	20.7025	0.3507	-0.3343	1.0440	0.9546
5	50.2189	0.4117	0.1627	0.5402	0.7268
6	26.6772	0.4426	0.0061	0.8441	0.8651
7	36.0158	0.2312	0.1983	0.9016	0.5831
8	41.2197	0.3426	0.1427	0.8891	0.5600
9	20.4477	1.2535	0.0102	1.0813	0.8273
10	30.0645	-0.1104	-0.1906	0.8703	0.7629
11	28.7403	-0.5980	-0.1428	0.8353	0.8064
12	14.5124	0.2234	-0.4502	1.0296	1.1635
All	337.7068	0.2286	0.0972	0.9496	0.6907

Table S4. Results of 2D Gaussian fitting. We fitted a 2D Gaussian model to the spatial distributions of gaze positions during the full contrast induction phases for each individual subject (marked as 1~12), and to the data pooled from all the subjects (marked as "All"). Here, x_0, y_0 showed the positions of the fits (unit: degree), σ_x, σ_y showed the widths of the fits (unit: degree), and A was the amplitude of the fit.
